# Caudate nucleus-dependent navigational strategies are associated with increased use of addictive drugs

**DOI:** 10.1002/hipo.22187

**Published:** 2013-10-25

**Authors:** Veronique D Bohbot, Daniel Balso, Kate Conrad, Kyoko Konishi, Marco Leyton

**Affiliations:** 1 Douglas Mental Health University Institute, Department of Psychiatry, McGill UniversityVerdun, Quebec, Canada; 2 Department of Psychiatry, McGill UniversityMontreal, Quebec, Canada

**Keywords:** tobacco, cannabis, hippocampus, spatial memory, response learning

## Abstract

This study aimed to investigate the relationship between navigational strategies and the use of abused substances in a sample of healthy young adults. Navigational strategies were assessed with the 4-on-8 virtual maze (4/8VM), a task previously shown to dissociate between hippocampal-dependent spatial navigational strategies and caudate nucleus-dependent stimulus-response navigational strategies. Spatial strategies involve learning the spatial relationships between the landmarks in an environment, while response learning strategies involve learning a rigid set of stimulus-response type associations, e.g., see the tree, turn left. We have shown that spatial learners have increased gray matter and fMRI activity in the hippocampus compared with response learners, while response learners have increased gray matter and fMRI activity in the caudate nucleus. We were interested in the prevalence of use of substances of abuse in spatial and response learners because of the evidence that people who score high on traits such as novelty seeking, sensation seeking, reward seeking, and impulsivity, are more cue-responsive and more likely to use substances of abuse. Since response learners show increased activity and gray matter in the caudate nucleus of the striatum, which is a brain area involved in addiction, we hypothesized that response learners would have a greater use of abused substances than spatial learners. Fifty-five young adults were tested on the 4/8VM and completed a time-line follow-back assessment of drug and alcohol use. We found that response learners had smoked a significantly greater number of cigarettes in their lifetime than spatial learners, were more likely to have used cannabis, and had double the lifetime alcohol consumption. We discuss the possible relationship between substance abuse and response strategies as well as the implications for the hippocampus, risks of neurological and psychiatric disorders, and healthy cognition. © 2013 The Authors. Hippocampus Published by Wiley Periodicals, Inc.

## INTRODUCTION

Humans and non-human animals use multiple memory systems that involve distinct brain structures when they navigate in the environment (O'Keefe and Nadel, 1978). The hippocampus is critical for allocentric spatial learning and memory, and the formation of a cognitive map, i.e. learning and memory for the relationships between environmental landmarks irrespective of the position of the observer, such that any target location can be reached in a direct path from any starting position (Scoville and Milner, 1957; O'Keefe and Nadel, 1978; Eichenbaum et al., 1990; Bohbot et al., 1998). The striatum, in comparison, is critical for response learning and memory, and habit formation by making rigid stimulus-response associations (Packard et al., 1989; McDonald and White, 1993; McDonald and White, 1994; Alvarez et al., 1995; Packard and McGaugh, 1996; Wolbers et al., 2004). The hippocampus and striatum are also involved in decision making processes (van der Meer et al., 2010). The decision making process dependent on the hippocampus, involves projecting one-self into future situations to create expectations about action outcomes. In contrast, the decision making process dependent on the striatum, uses past experiences to associate actions with values.

It is suggested that these systems function in a cooperative fashion (Lansink et al., 2009), independently (Bohbot et al., 2004; Mizumori et al., 2004) and competitively (Packard, 1999). In a dual-solution task, which allows both spatial and response navigational learning strategies, studies in rodents showed increased basal levels of acetylcholine in the hippocampus of rodents prior to the spontaneous use of the spatial strategy. In comparison, rodents that spontaneously used a response strategy in the dual solution task had increased basal levels of acetylcholine in the striatum (Chang and Gold, 2003). Similarly, we found that young adults who navigated using a spatial strategy showed greater fMRI activity (Iaria et al., 2003) and gray matter (Bohbot et al., 2007) in the hippocampus. Conversely, young adults who navigated using response strategies showed increased fMRI activity (Iaria et al., 2003) and gray matter (Bohbot et al., 2007) in the caudate nucleus of the striatum.

The use of spatial strategies decreases across the lifespan (Barnes et al., 1980; Rapp et al., 1997; Bohbot et al., 2012; Etchamendy et al., 2012; Rodgers et al., 2012). Whereas ∼90% of 7- to 8-year olds use spatial learning strategies, this drops to fewer than 40% in adults in favor of response learning strategies, perhaps as early as age 17 (Bohbot et al., 2012; Lin et al., 2012). This shift from spatial to response strategies may be a biologically adaptive mechanism that is hypothesized to be associated with three potential factors: repetition that normally occurs during the formation of habits, stress and reward. Each will be discussed in turn.

Response strategies are efficient when navigating in an environment where the start and target locations are constant, as in route learning paradigms (Hartley et al., 2003; Iaria et al., 2003; Head and Isom, 2010). This increased efficiency is reflected by a reduction in latencies and errors to find target locations (Iaria et al., 2003) and it results from the repetition of traveling from a constant start to target location. In fact, it has been shown that rodents trained to find a target location on a plus maze initially use spatial strategies, but then after a few days adopt a response strategy. Therefore, repetition of a behavior leads to a shift from hippocampus-based spatial strategy to a caudate-nucleus based response strategy (Packard and McGaugh, 1996; Gold, 2004).

In contrast to the way response strategies can be efficient as described above, response strategies can also be inefficient when the relationship between the start and target position changes and a novel path must be derived (Hartley et al., 2003; Driscoll et al., 2005). Spatial strategies, which involve building relationship between environmental landmarks to form a cognitive map, now become more efficient as they enable deriving novel paths. In summary, response strategies are more efficient when there are constant start and target locations, evidenced by faster latencies and error reduction. However, response strategies are less efficient when a new pathway needs to be derived, because in the absence of a cognitive map, the individual gets lost with novel start and target locations. The drive toward efficiency may be an important underlying factor behind the shift in strategies across the life span. With the repetition of a successful behavior and a constant start and target location, a response strategy emerges, leading to the automatization of behavior or habit formation (Iaria et al., 2003). However, this shift toward response strategies comes at a cost when a novel path must be derived requiring a cognitive map to navigate successfully.

Other lifestyle factors can produce a shift from using spatial strategies to response strategies. For example, stress and addiction related rewards, have been shown to affect the integrity of the hippocampus. Stress was reported to impair the hippocampus through the actions of glucocorticoids (Sapolsky et al., 1990; Sapolsky, 1994; McEwen and Sapolsky, 1995; Conrad et al., 1996; McKittrick et al., 2000; Kleen et al., 2006) and was shown to have an effect on navigational strategies. Schwabe et al. (2007,2008, 2012) found that chronic stress, acute stress, and prenatal stress can also increase the use of response strategies in rodents and humans tested on a navigation task. Interestingly, stress has also been shown to shift goal-directed behaviors to habitual actions that are dependent on the striatum (Schwabe et al., 2011). Addiction related rewards such as tobacco (Piri et al., 2012), opiates (Lu et al., 2010), psychostimulants (Arias-Cavieres et al., 2010) and alcohol (Welch et al., 2013) all have been shown to have negative effects on the hippocampus.

Response learning and various forms of impulsivity are associated with cue sensitivity (Gray, 1987). For example, response learning is based on stimulus-response associations where stimuli act as cues. As well, aspects of impulsivity, such as behavioral disinhibition, sensation seeking and novelty seeking, are increased in human drug users (Tarter et al., 2003; Milivojevic et al., 2012; Castellanos-Ryan et al., 2013; Pingault et al., 2013), while rodent analogs of these traits are thought to include heightened cue-sensitivity (Flagel et al., 2010–2011; Saunders and Robinson, 2010; Saunders and Robinson, 2011; Belin and Deroche-Gamonet, 2012). Moreover, impulsivity, cue sensitivity and susceptibility to substance abuse are all associated with increased striatal gray matter (Ersche et al., 2011; Groman et al., 2013). As the use of response strategies over spatial strategies is associated with more gray matter in the caudate nucleus of the striatum, research in the current paper investigates whether response learners are more susceptible to using substances of abuse, as compared with spatial learners. To establish a relationship between drug use and navigation strategies we tested a large group of healthy young adults with no substance use disorders on a virtual navigation task that can be solved using either a spatial or response learning strategy and assessed their lifetime drug consumption.

## METHODS

### Participants

Sixty healthy young adult participants (mean age = 21.4, SD = 2.56, 36 women, 24 men) entered in the study. An extensive phone questionnaire was administered to screen for past history of psychiatric or neurological disorders. The questionnaire includes various components such as demographic information, vision, motion sickness, medical history, cardiovascular diseases, neurological disorders, medical conditions, psychiatric disorders, substance abuse, general medication, family history, and handedness. Participants were asked whether they have ever been treated for using drugs or alcohol abusively to exclude for any substance use disorders. Fifty-five participants completed the study; one was excluded for past history of psychiatric illness and four others were excluded due to incomplete data. Of the included participants six were left-handed. Testing occurred at the Memory and Motion Laboratory at the Douglas Mental Health University Institute. Participants volunteered for the testing session after being recruited through word of mouth. Informed consent was obtained in conformity with the local ethics committee requirements.

### Task

**Figure 1:**
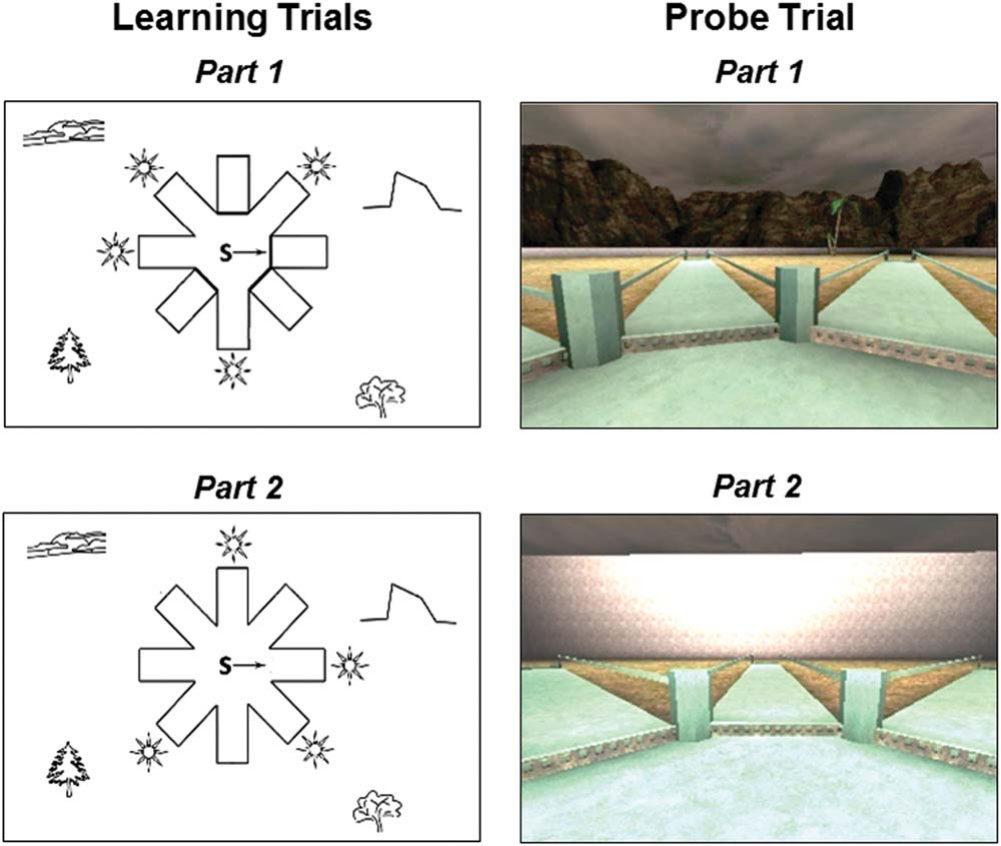
Schematic drawings and first person views of the four on eight virtual maze (4/8VM) learning trials (left) and probe trial (right). The 4/8VM is an eight-arm radial maze that is surrounded by both distal and proximal landmarks: two trees, mountains, and a sunset. Parts 1 and 2 administered during the learning trials are situated in the environment depicted in the top right picture. The top left diagram shows a top view of part 1 of the learning trials, where four arms are blocked and four arms are open. Bottom left diagram shows a top view of part 2 of the learning trials, where all arms are open. Part 1 of the probe trial is the same as part 1 of the learning trials. In part 2 of the probe, all landmarks are removed and a wall is raised around the environment, as depicted in the bottom left picture.

The 4-on-8 Virtual Maze (4/8VM) is a virtual reality task that was created using programming software from a commercially available computer game (Unreal Tournament; Epic Games, Raleigh, NC) (Fig. 1). Before beginning the task participants practiced navigating using a standard keyboard in a similar but landmark-free virtual environment to practice the motor aspects of the task. The 4/8VM consists of an eight arm radial maze situated in an enriched environment. The environment contains distal landmarks: two trees, mountains, and a sunset. At the end of each arm there are stairs that lead to a small pit where, in some cases, a participant can pick up an object. The participant is always unable to see the objects from the center of the maze.

The task consists of five trials where each trial has a Part 1 and a Part 2. In Part 1, a set of barriers block four of the eight arms. The participant is instructed to pick up objects located at the end of the four open arms. Additionally, the participant is told to remember where they visited because in Part 2, all of the arms are accessible and the objects are situated in the arms that were previously inaccessible. All landmarks are visible during Part 1 and Part 2 of the first three trials or until participants reach a criterion of no errors on part 2 of a single trial. This criterion ensures that all participants have learned the task.

Once this criterion is reached, a probe trial is administered. During Part 1 of the probe trial the participants still collect the objects from the open arms and all landmarks are present, however, in Part 2 when all of the arms are accessible, a wall is erected around the maze so that the participants cannot see the environment. Participants can solve the 4/8VM using either of two strategies. The first, a “spatial” strategy, depends on the relationship between the objects and the environment. For example, a participant would remember the position of an object relative to the trees and the mountain. The second is a “response” strategy where a counting or patterning system is used to remember the sequence of rewarded arms. The probe trial does not disturb the performance of participants using a response strategy as their sequence does not depend on the environmental landmarks. Conversely, participants using a spatial strategy have difficulty on Part 2 of the probe trial because they require the landmarks to properly retrieve the objects. Therefore, Part 2 of the fourth trial is considered a probe trial because it differentiates participants who use a spatial strategy from those who use a response strategy. For example, participants who rely on the landmarks in the environment will be more likely to make errors when the landmarks are removed (Iaria et al., 2003). The last trial again consists of a Part 1 and a Part 2; however, landmarks are visible in both parts. This trial allows us to assess whether participants who use a spatial strategy during the first three trials will switch to a response strategy after the probe.

At the end of the task participants were debriefed. They were asked to report how they knew which pathways contained objects and which were empty in the Part 2 trials. Based on their description, participants were categorized in either a spatial strategy group or a response strategy group.

### Questionnaire

Participants also completed a time-line follow-back assessment of lifetime drug and alcohol use. Drugs assessed included tobacco, cannabis, cocaine, amphetamine, psilocybin, methylenedioxymethamphetamine (MDMA), ketamine, gamma-hydroxybutyric acid (GHB), ephedrine, steroids, opiates, and lysergic acid diethylamide (LSD). Beside each listed substance of abuse, participants were asked to note whether or not they had used the drug and the number of times they used that drug throughout their lives.

## RESULTS

Fifty-five participants were tested on the 4/8VM and were given the questionnaire on substance use. Subjects were divided into two groups based on their initial strategy on the 4/8VM. Strategy was assessed according to verbal reports. A total of 78.2% (*N* = 43) of the participants spontaneously employed a response strategy while 21.8% (*N* = 12) used a spatial strategy. Although not significant, spatial learners (mean = 0.67) made slightly more probe errors than response learners (mean = 0.42) (*t* = −1.1, *P* = 0.275). Both spatial and response learners had similar latencies and errors throughout the learning trials (Latency: *F* = 0.489, *P* > 0.05; Errors: *F* = 0.223, *P* > 0.05). Spatial and response learners did not differ in age (spatial = 20.08 ± 1.38, response = 21.72 ± 2.83; *t*_(54)_ = 1.931, *P* > 0.05), or IQ measured by the Shipley IQ test (spatial = 110.67 ± 4.68, response = 106.65 ± 8.75; *t*_(54)_ = −1.52, *P* > 0.05).

**Figure 2:**
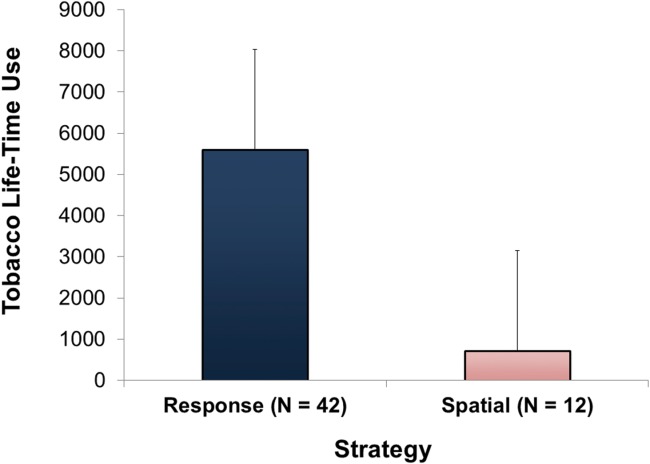
Response learners consume more tobacco than spatial learners: Response learners (*N* = 43) consumed significantly more cigarettes (mean = 5600 ± (SEM) 2301.9) throughout their lives compared to spatial learners (*N* = 12; mean = 714.17 ± (SEM) 510.76) (*t*_(52)_ = −2.072; *P* < 0.05). Tobacco data for one participant was unavailable. The large variability stems from the fact that both groups include users and non-users. [Color figure can be viewed in the online issue, which is available at http://wileyonlinelibrary.com.]

Results from the drug-use questionnaire demonstrated that the groups had different lifetime patterns of tobacco use. Response learners (*N* = 42; mean = 5600.21 ± 14918.24) consumed significantly more cigarettes than spatial learners (*N* = 12; mean = 714.17 ± 1769.32) throughout their lives (*t*_(52)_ = −2.072; *P* < 0.05) (Fig. 2). The large standard deviation values reflect considerable variability in the data. A close inspection of the data indicates two subgroups within the tested population. A subgroup of the population are non-smokers and therefore have very low values, while another subgroup are heavy smokers and have high lifetime use values. Because there were two subgroups with regards to tobacco use (i.e. smokers and non-smokers), we performed a chi-square analysis, which was not significant (χ^2^ = 1.043, *P* = 0.307).

**Figure 3:**
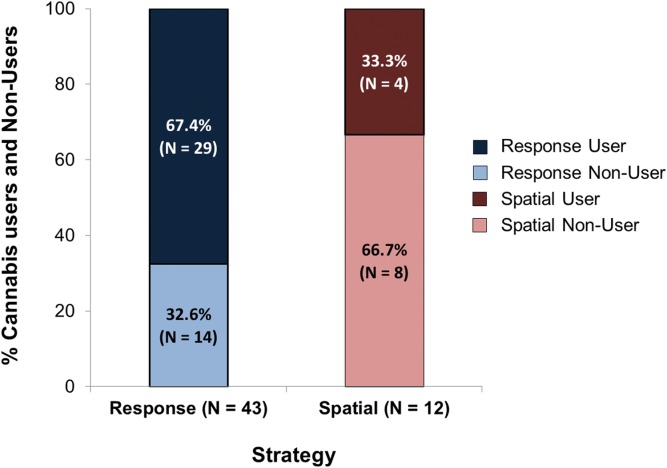
A higher proportion of cannabis users in response learners: A chi-square analysis examining cannabis users and non-users in each navigational strategy group showed that there was a significantly higher proportion of non-users in the spatial group (66.6%) compared with the response group (32.6%). In the response group, there was a higher proportion of cannabis users (67.4%) compared with non-users (32.6%) (× 2(53) = 4.55; *P* < 0.05). [Color figure can be viewed in the online issue, which is available at http://wileyonlinelibrary.com.]

A chi-square analysis examining the proportion of cannabis users and non-users in each strategy group showed that there were a significantly higher proportion of non-users in the spatial group compared with the response group (Fig. 3). Response learners had a higher proportion of cannabis users (67.4%) than non-users (32.6%) while spatial learners had a higher proportion of non-users (66.7%) than users (33.3%) (*x*^2^_(53)_ = 4.55; *P* < 0.05). Furthermore, we found a correlation between tobacco and cannabis use (*r* = 0.62, *P* < 0.001). Participants were classified as a user if they had more than one lifetime use of cannabis. Participants with one or zero lifetime use of cannabis were considered non-user. There was no significant difference in strategy (χ^2^ = 0.37, *P* = 0.85) and performance (errors, latency, probe: *P* > 0.05) between participants with one or zero lifetime use of cannabis.

Response learners tended to have higher lifetime incidence rates of alcohol intoxication compared with spatial learners (Response mean = 201.97 ± 335.56, Spatial mean = 89.3 ± 100.07; *t* = 1.041, *P* = 0.303). However, discrepancies were found within the data concerning the interpretation of alcohol intoxication in the questionnaire. Some individuals interpreted the question as alcohol intoxication requiring a hospital visit, while others interpreted the question as inebriation. Therefore, values provided for alcohol intoxication may be underestimated for certain individuals. Alcohol data were missing for nine participants. Nevertheless, the average rate of intoxication among response learners was double that of spatial learners.

**Figure 4:**
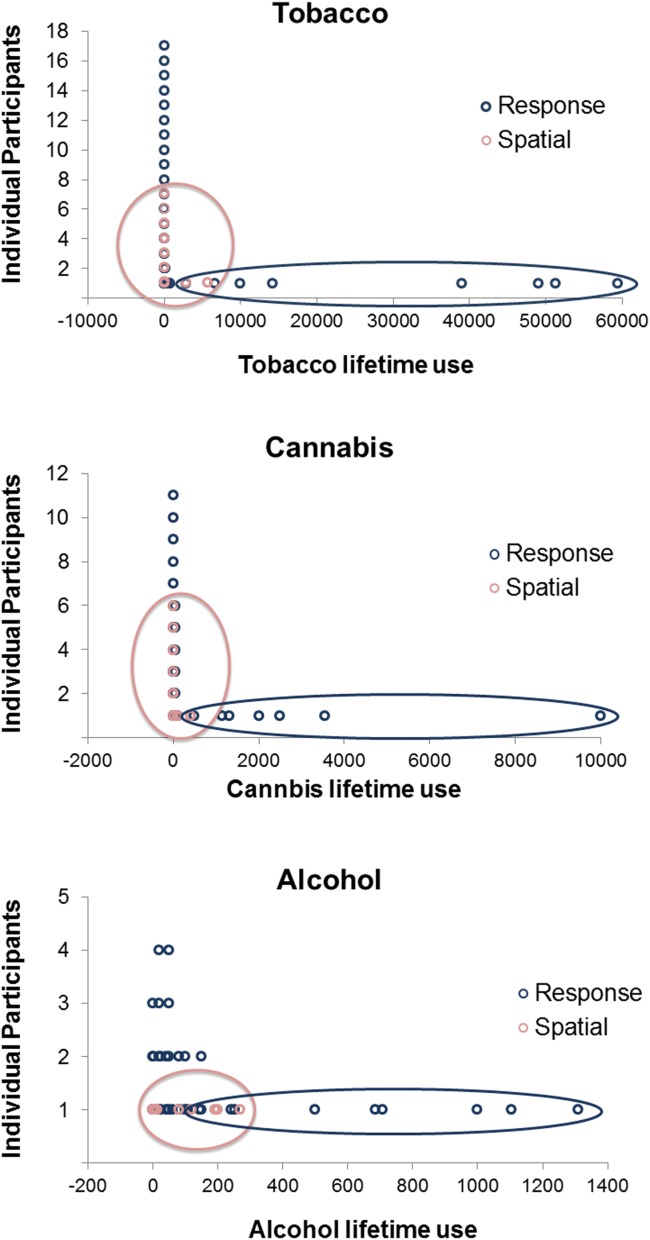
Distribution of spatial and response learners' lifetime use of tobacco (top), cannabis (middle), and alcohol (bottom). Individual participants are lined up vertically for each substance used to display the distribution of individual participants. Therefore, a higher number of participants is reflected in a higher number of circles that are line up vertically. The highest circle represents the total number of subjects for that use of substance. The red ellipses surround the distribution of spatial learners. As can be seen in the figure, the distribution of spatial learners revolves around the zero consumption of all three substance of abuse use. Although a number of response learners do not consume substances of abuse, it is apparent that most of the people who are consumers (indicated by the green ellipse) are in the response learning group. [Color figure can be viewed in the online issue, which is available at http://wileyonlinelibrary.com.]

No other statistical analyses were carried out for the remaining drugs of abuse because usage within the tested population was too minimal. While cannabis and tobacco involved 33 and 25 users, respectively, cocaine, amphetamine, psilocybin, and MDMA involved 10 users or less (Table1). Furthermore, ketamine, GHB, ephedrine, steroids, opiates, and LSD had fewer than five users. Similar spatial/response distributions were observed for alcohol (Fig. 4, bottom), cannabis (Fig. 4, middle), nicotine (Fig. 4, top), cocaine, amphetamine, psilocybin, and MDMA. The other drugs (ketamine, GHB, ephedrine, steroids, opiates, and LSD) did not have enough users to display a distribution. All substances users were more likely to be response learners.

**Table 1:** Substances of abuse: total lifetime uses, total number of subjects, and total number of users.

	Spatial		Response	
		*Total # of subjects*		*Total # of subjects*
Substances of abuse	*Total lifetime uses (mean ± SD)*	*Number of users*	*Total lifetime uses (mean ± SD)*	*Number of users*
**Alcohol**	893 (89.3 ± 100.07}	*N* = 10	7,271 (201.97 ±335.56)	*N* = 36
		*N* = 9 (90%)		*N* = 33 (92%)
**Cannabis**	593 (49.42 ± 115.76)	*N* = 12	21,697 (504.58 ± 1,656.89)	*N*=43
		*N* = 4 (33%)		*N* = 29 (67%)
**Tobacco**	8,570 (714.17 ± 1,769.32)	*N* = 12	235,209 (5,600.21 ± 14,918.24)	*N*=43
		*N*=4 (33%)		*N* = 21 (49%)
**Cocaine**	6 (0.5 ±1.73)	*N* = 12	**370** (8.6 ± 45.83)	*N*=43
		*N* = 1 (8%)		*N* = 6 (14%)
**Amphetamine**	3 (0.25 ±0.87)	*N* = 12	89 (2.07 ± 8.23)	*N*=43
		*N* = 1 (8%)		*N* = 5 (12%)
**Psilocybin**	2 (0.17 ±0.58)	*N* = 12	23 (0.53 ± 1.24)	*N*=43
		*N* = 1 (8%)		*N* = 7 (16%)
**MDMA**	10 (0.83 ± 2.89)	*N* = 12	116 (2.70 ± 10.19)	*N*=43
		*N* = 1 (8%)		*N* = 6 (14%)
**Ketamine**	0 (0 ± 0)	*N* = 12	10 (0.23 ± 1.52)	*N*=43
		*N* = 0 (0%)		*N* = l (2%)
**GHB**	1 (0.08 ±0.29)	*N* = 12	14 (0.33 ± 1.58)	*N*=43
		*N* = 0 (0%)		*N* = 2 (5%)
**Ephedrine**	0 (0 ± 0)	*N* = 12	0 (0 ± 0)	*N*=43
		*N* = 0 (0%)		*N* = 0 (0%)
**Steroids**	0 (0 ± 0)	*N* = 12	3 (0.07 ±0.46)	*N*=43
		*N* = 0 (0%)		*N* = 1 (2%)
**Opiates**	0 (0 ± 0)	*N* = 12	0 (0 ± 0)	*N* = 43
		*N* = 0 (0%)		*N* = 0 (0%)
**LSD**	0 (0 ± 0)	*N* = 12	2 (0.05 ±0.21)	*N* = 43
		*N* = 0 (0%)		*N* = 0 (0%)

* Users>1 use; non-users ≤ 1 use.

## DISCUSSION

This study investigated the prevalence of use of substances of abuse in healthy young adults in relation to navigational strategies measured with the 4/8VM task. We found that response learners, compared with spatial learners, reported significantly more use of substances such as tobacco and cannabis and had double the lifetime consumption of alcohol. Therefore, as hypothesized, the prevalence of use of substances of abuse was high in response learners. A longitudinal study will be needed to establish the causal relationship between response strategies and drug use. In other words, the use of response strategies early in life could predispose an individual to drug use, and conversely, drug use could increase the probability of using of response strategies. It is most likely a combination of both and we provide evidence for the two mechanisms below. Importantly, this is the first study to demonstrate an association between use of drugs of abuse and the use of response strategies in a dual solution task.

In support of the theory that strategy may influence use of drugs of abuse, previous studies showed that response navigational strategies emerge as early as age 7 years old (Bohbot et al., 2012; Lin et al., 2012), well before the onset of using abused substances. In other words, cue sensitive response learners (measured with our virtual navigation task, the 4/8VM) may also be more responsive to reward cues, making them more susceptible to trying and using drugs.

Substances of abuse may also promote the use of caudate nucleus-based response learning through their stimulation of the striatum (White, 1996). In turn, people who regularly engage in striatal dependent reward seeking behavior may be more likely use response learning strategies when navigating. Specifically, dopamine release has been measured in the caudate nucleus of participants not only exposed to amphetamines (Boileau et al., 2007), cocaine (Cox et al., 2009), and alcohol (Barrett et al., 2008), but also to other types of rewards, which are more common such as chocolate (Small et al., 2001) and video games (Koepp et al., 1998; Erickson et al., 2010). In fact, video game users, engaging in only 9 h of video games per week or more, were shown to have a significantly larger striatum (including the nucleus accumbens and caudate nucleus) (Kuhn et al., 2011). In another study gray matter in the striatum at baseline predicted the level of video game skill acquired by the research participants (Erickson et al., 2010). In this study, gray matter in the hippocampus had no impact on participant's ability to acquire skills with the video game. Furthermore, studies that specifically investigated superior visuospatial performance in action video game experts demonstrated that this skill was not correlated with gray matter in the hippocampus, but rather with gray matter in the right posterior parietal cortex (Tanaka et al., 2013). In another study, the volume of the entorhinal cortex, which sends afferent projections to the hippocampus, negatively correlated with playing action video games (Kuhn and Gallinat, 2013) suggesting that the entorhinal cortex shrinks with time playing action video games. Interestingly, the same region of the brain positively correlated with time playing logic and puzzle games. These results are consistent with the hypotheses derived from a study by Foerde and Shohamy (2011) showing that delaying feedback timing will shift from learning based on the striatum to hippocampus. Therefore, in the case of video games, the immediate rewards present in action video games would stimulate the striatum to grow and the entorhinal cortex to shrink, whereas the delayed rewards present in puzzles or logic games would stimulate the entorhinal cortex to grow (Foerde and Shohamy, 2011; Smith-Dijak et al., 2013; Shohamy and Adcock, 2010). While a region of interest analysis shows a correlation between a composite measure of time playing video games and grey matter in the hippocampus, Kuhn and Gallinat (2013) did not report whether the hippocampus also correlated negatively to action video games, as did the entorhinal cortex. Interestingly, consistent with the findings of the current paper, the authors did report a significant correlation between time playing video games and both internet addiction and alcohol consumption. Therefore, reward seeking behavior may engage the striatum which in turn would promote response navigational strategies. Since reward seeking behavior can be expressed at a very young age in childhood (for example in the form of a child seeking food rewards like chocolate, or material rewards such as toys, games, and playing video games), reward seeking behavior in childhood may also stimulate the striatum and precede the onset of response navigational strategies which were measured as early as 7 years of age in ∼5% of the children tested by Lin et al. (2012). In summary, reward seeking behavior at a very young age could engage the striatum and response learning, and predispose to drug seeking behavior.

There is a transition from voluntary use to involuntary compulsive use of substances of abuse (Schwabe et al., 2011). Voluntary use is dependent on the prefrontal cortex and dorsomedial striatum and involves goal-directed behaviors. This behavior is driven by the hedonic effects of the drugs and/or rewards. In contrast, involuntary use depends on the dorsolateral striatum and involves habitual action that are independent of the expected outcome (Everitt et al., 2001; Everitt and Robbins, 2005). Dopamine is involved in the development of habitual actions, and as mentioned earlier, is increased in the striatum after drug use. Habitual response to drugs of abuse have especially been shown to develop remarkably fast (Dickinson et al., 2002; Miles et al., 2003). As such, drug use can lead to a fast transition from goal-directed behavior to habitual action and drug addiction.

Since response learners show less activity and gray matter in the hippocampus than spatial learners (Iaria et al., 2003; Bohbot et al., 2007), the current results may also reflect an impact of drug use on hippocampal based spatial strategies. Interestingly, acute nicotine has been shown to positively affect certain cognitive domains (Swan and Lessov-Schlaggar, 2007). In addition, several rodent studies have shown that differential doses of nicotine facilitates spatial memory (Socci et al., 1995; Abdulla et al., 1996; Bernal et al., 1999), while others have shown that chronic nicotine exposure negatively affects spatial memory and hippocampal morphology (Scerri et al., 2006). Therefore, nicotine may have positive effects on spatial memory with acute administration, but detrimental effects under chronic administration. In our study, participants were neither given nor deprived of tobacco. Therefore, the potential effects of tobacco on navigational strategies result from chronic consumption and do not result from acute effects of tobacco on non-smokers, or withdrawal effects in smokers. Consistent with previous studies, chronic tobacco consumption was detrimental to spatial memory strategies in the current study.

Abrous et al. (2002) taught rats to self-administer nicotine at similar levels to those of a heavy smoker. They found that nicotine had several deleterious effects on the dentate gyrus of the hippocampus: decreased neurogenesis, decreased expression of a cell adhesion protein associated with cell migration, and increased cell death. Their results provide a physiological explanation for deficits in hippocampal based tasks seen in rodents. For example, Scerri et al. (2006) found that rats given high doses of nicotine had impairments learning a spatial task such as the Morris Water Maze. The same group reported less neurogenesis in the dentate gyrus of rats given high doses of nicotine compared with rats given low doses of nicotine and nicotine naive rats. In human smokers, chronic nicotine exposure is associated with a lower concentration of an axonal cell marker in the hippocampus when compared with non-smokers (Gallinat et al., 2007). Although nicotine is a main constituent of tobacco smoke, humans inhale countless numbers of other toxic substances while smoking. These substances could also affect the integrity of the hippocampus. Indeed, rats exposed to chronic cigarette smoke have neuropathological alterations in the hippocampus (Ho et al., 2012). Therefore, through affecting the morphology of the hippocampus, smoking could decrease the likelihood of using spatial learning strategies.

In addition, we found a correlation between tobacco and cannabis use and so it follows that response learners, who consumed a significant amount of cigarettes in their lifetimes, were more likely to smoke cannabis than spatial learners. However, aside from cigarette smoking, smoking cannabis may also affect the use of spatial strategies due to its impact on the hippocampus and spatial memory. This impact is noted in rodent as well as human literature. Rubino et al. (2009) found that adolescent rats pretreated with tetrahydrocannabinol (THC), the active substance contained in cannabis, show impaired spatial memory in adulthood, measured by a radial maze task. The same group also found morphological changes in the dentate gyrus of THC pretreated rats. Further, heavy cannabis use negatively correlated with gray matter in human hippocampus (Yucel et al., 2008) while moderate cannabis use was associated with reduced performance on a logical memory task, that is generally considered to be a measure of episodic memory (Indlekofer et al., 2009). Cannabis use has also been associated with decreased activity in the hippocampus during an associative memory task (Jager et al., 2007). In summary, cannabis use was shown to be detrimental to the hippocampus.

A recent study supports the hypothesis that substances of abuse have a negative impact on hippocampus-dependent spatial learning and promotes caudate-nucleus dependent response learning. Researchers looked at the impact of reward processing on spatial and response learning in mice: drug (morphine)-rewarded mice performed significantly better than control mice on a cue discrimination task that involved stimulus-response learning. In contrast, drug-rewarded mice were significantly impaired in a spatial task compared with control mice (Baudonnat et al., 2011). The group also saw an altered expression pattern of phosphorylated CREB, a transcription factor associated with learning, in drug reinforced mice: CREB was expressed in the striatum of mice irrespective of the task (cue or spatial). Control mice showed CREB expression in the striatum or hippocampus depending on whether they leaned the cue task or spatial task, respectively. Therefore, drug reinforcement negatively affected hippocampal learning in favor of response learning (Gabriele et al., 2009). These results combined with the toxic effects of drugs on hippocampal integrity may have adverse long term effects on cognition.

**Figure 5:**
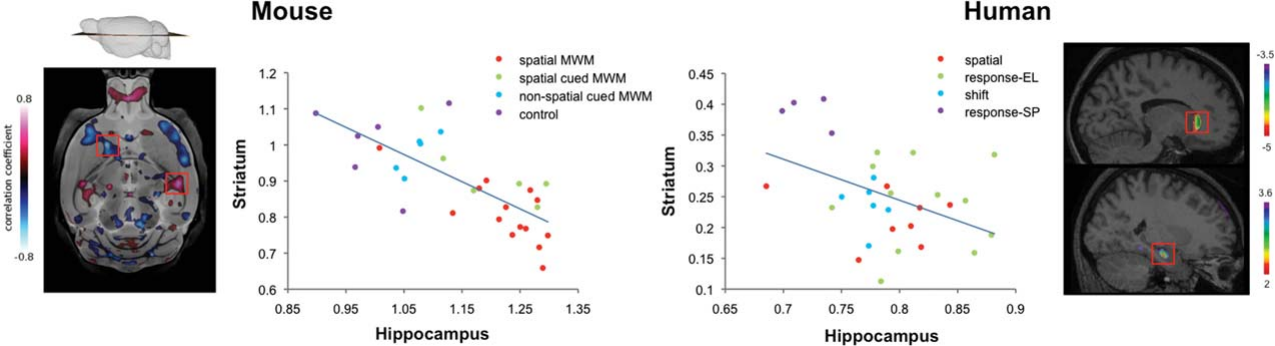
Inverse relationship between hippocampus and caudate nucleus gray matter in mice (left) and humans (right). The mouse MRI image depicts a correlation between hippocampus CA1 gray matter (taken from a region near the lower red square) and whole brain gray matter, indicating a negative correlation with the striatum (identified with the top red square). Mice were trained for 5 days on one of four different versions of the Morris water maze: spatial MWM (standard spatial memory version of the Morris water maze task), spatial cued MWM (version involving a cue over the target platform and visible extra-maze landmarks), non-spatial cued MWM (version with a cue over the target platform and a curtain around the maze hiding extra-maze landmarks), and controls (mice which were handled but did not receive training). Non-spatial mice in this task are engaged in a stimulus-response strategy. Using high-resolution MRI, authors revealed a negative correlation between gray matter in the hippocampus and striatum in all mice. In other words, mice with more gray matter in the hippocampus have less gray matter in the striatum and vise versa. The mouse MRI image and data are courtesy of Dr. Jason Lerch, Mouse Imaging Centre, Hospital for Sick Children, Toronto, Ontario, Canada and was published in Lerch et al. (2011). The human MRI images depict regression analyses between errors made by young adult participants while performing the 4/8VM probe trial (when all landmarks were removed) and whole brain gray-matter density. Gray matter in the hippocampus positively correlated with probe errors while gray matter in the caudate nucleus negatively correlated with probe errors. Images show the results superimposed onto an anatomical MRI and are displayed in the sagittal plane. There was a negative correlation between gray matter in the hippocampus at peak (*x* = 24, *y* = 13, *z* = 20) and gray matter in the caudate nucleus at peak (*x* = 14, *y* = 28, *z* = 4) (indicated with the red squares: top for caudate nucleus and bottom for the hippocampus). Spatial and Response navigational strategy groups depicted are further divided into the following groups: spatial learners who maintain using spatial strategies with training (spatial), spatial learners who shift to using a response strategy with training (shift), response learners who used a pattern of open and closed pathways from an external landmark such as a tree (response-EL), and response learners who used a pattern of open and closed pathways from the starting position (response-SP). Human MRI images were adapted from a previous publication (Bohbot et al., 2007).

Data showing a concurrent impairment of spatial memory and promotion of response learning in drug-taking mice (Baudonnat et al., 2011) are consistent with the observation of an inverse relationship between gray matter in the caudate nucleus of the striatum and hippocampus, in both humans (Bohbot et al., 2007) and rodents (Lerch et al., 2011). In the human study, participants' MRIs were regressed against a measure of spatial strategies with voxel based morphometry (Fig. 5). Results showed a positive correlation between spatial memory and gray matter in the hippocampus and a negative correlation with the caudate nucleus of the striatum. Importantly, gray matter in the hippocampus and caudate nucleus were negatively correlated such that an increase in gray matter in one area was associated with a decreased gray matter in the other (Bohbot et al., 2007). In rodents, a prospective training study showed that mice trained on a spatial memory version of the Morris water task had a significant increase in gray matter in the hippocampus, in contrast to the mice trained on the stimulus-response version of the task, whereby they learned to swim to a visibly identified platform (Lerch et al., 2011). Importantly, after only five training days, significant morphological changes occurred, providing evidence for the fact that experience has a significant impact on gray matter. As in the human MRI study, gray matter in the hippocampus and striatum of trained mice were negatively correlated (Fig. 5). These data suggest that using the caudate nucleus-dependent response strategy comes at a cost for the hippocampus-dependent spatial strategy. As argued earlier, reward seeking behaviors may be present at a very young age in childhood, thereby promoting the caudate nucleus at a cost for the hippocampus very early on in life. In fact, response strategy users exhibit a decrease in hippocampus to striatal volume from age 8 to 18 while spatial strategy users exhibit an increase in hippocampus to striatal volume from childhood to adolescence (Lin et al., 2012). Consequently, the inverse relationship between the caudate nucleus of the striatum and the hippocampus was found in rodents and in humans, in terms of gray matter (Fig. 5) and function (Packard and McGaugh, 1996; Iaria et al., 2003), suggesting that reward seeking behavior, possibly in childhood, can promote the striatum at the expense of the hippocampus.

It is well established that substances of abuse have a negative impact on hippocampal integrity and function; however, the reverse relationship may also be true: an impaired hippocampus could also increase vulnerability to substance use and addiction. In a rodent study, suppression of adult hippocampal neurogenesis increased vulnerability to addiction and increased resistance to extinction in rats trained to self-administer cocaine (Noonan et al., 2010). Stress is a factor known to suppress hippocampal neurogenesis (Tanapat et al., 2001). Rodents experiencing great amounts of stress by being exposed to a predator's smell, in this case a fox, were shown to have significantly reduced neurogenesis in the hippocampus. Therefore, early stress may have an impact on the hippocampus, which in turn will increase vulnerability to substance abuse. Mothers of response strategy users reported having experienced significantly more stressful events during pregnancy compared with mothers of spatial strategy users (Schwabe et al., 2012). In fact, even genetic differences have been shown to be associated with the use of response strategies (Bueller et al., 2006; Banner et al., 2011). In summary, an impaired hippocampus will increase vulnerability to substance abuse. Engagement in substance abuse early in life, or later in life, will further increase stimulation of the striatum at the expense of the hippocampus.

Conversely, a number of studies have looked at the protective effect of exercise on rodent models of drug addiction (McMillan et al., 1995; Cosgrove et al., 2002; Smith et al., 2008; Smith and Pitts, 2011) because voluntary wheel running (considered exercise) is associated with increased neurogenesis in the dentate gyrus of the hippocampus (van Praag, 2008). For example, Smith and Pitts (2011) showed that voluntary wheel running before drug self-administration reduced acquisition and levels of active drug seeking behaviors when compared with the behavior of rodents who were not allowed to exercise. Similar to rodents, exercise increases the size of the hippocampus in humans (Erickson et al., 2011), and exercise attenuates cigarette cravings and withdrawal symptoms in humans (Taylor et al., 2007). Since spatial learning strategies are associated with increased activity and gray matter in the hippocampus (Iaria et al., 2003; Bohbot et al., 2007), spatial learners could be less likely to exhibit reward seeking behaviors in a similar fashion to exercise. In fact, spatial learning stimulates growth in the hippocampus, beyond the effects of exercise (Lerch et al., 2011).

To date, there are few longitudinal studies in humans that associate brain areas with vulnerability to addiction. Cheetham et al. (2012) reported that adolescents who started using cannabis had smaller orbitofrontal cortex (OFC) volumes prior to initiation of drug use than their non-drug using peers. The OFC is associated with impaired decision making in drug addiction (London et al., 2000) while an intact OFC plays an important role in spatial learning: an intact OFC is essential for acquisition and consolidation of spatial memory in rats (Vafaei and Rashidy-Pour, 2004) and it is involved in the retrieval of route related contextual information in humans (Brown et al., 2010). Consistent with these findings, repeated high-frequency transcranial magnetic stimulation (rTMS) of another region of the frontal cortex, the dorsolateral prefrontal cortex (DLPFC), reduced cigarette consumption (Amiaz et al., 2009; Feil and Zangen, 2010). Furthermore, spatial strategies assessed with the 4/8VM have been associated with increased gray matter in the OFC (Dahmani and Bohbot, 2013). Therefore, the OFC is involved with both reward based decision making and spatial navigation. Thus, decreased function of the OFC may not only impact an individual's propensity to seek rewards but may also decrease their likelihood of using a spatial navigation strategy.

It would also be of interest in future studies to examine navigational strategies in addicts and use this information to guide intervention strategies. For example, response strategy patients may be more responsive to extinction training, while spatial strategy patients may be more responsive to cognitively guided therapies.

In summary, we found that a greater proportion of response learners smoked cannabis and smoked a greater number of cigarettes in their lifetime than spatial learners. Response learners also had double the lifetime consumption of alcohol relative to spatial learners. Further, we found a positive correlation between smoking cigarettes and smoking cannabis. Cue-driven individuals may be more likely to use response navigational strategies and try and use drugs later in life.

Data in the literature also show that chocolate and video games stimulate dopamine release in the striatum. Video games are associated with a larger striatum, action video games are negatively correlated with grey matter in the entorhinal cortex and skills gained with video game playing are not associated with the hippocampus, but are associated with a larger striatum at baseline. Even the visuospatial superiority noted in action video game experts was not associated with gray matter in the hippocampus. Therefore, the vulnerability to substance abuse reported in this paper may be preceded by reward seeking behaviors in childhood such that individuals who seek rewards (foods such as chocolate, toys, games, video games etc.), also seek novelty and display response navigational strategies at the expense of hippocampus-based spatial memory strategies.

Additional research is required to elucidate the causal link between drug use and the use of response strategies. Numerous questions also remain concerning anatomical correlates between drug use and the use of response strategies. Although it is unclear as to whether the same regions of the striatum are involved in response learning and addiction, studies have shown an inverse relationship between the function and gray matter of the hippocampus and caudate nucleus of the striatum. Therefore regular reward stimulation may come at a cost and compromise the hippocampus. In turn, reduced gray matter in the hippocampus has been associated with an increased risk for numerous neurological and psychiatric disorders such as Schizophrenia (Pantelis et al., 2003), post-traumatic stress disorder (Gilbertson et al., 2002), depression (Amico et al., 2011) and Alzheimer's disease (Apostolova et al., 2006; Swan and Lessov-Schlaggar, 2007). Therefore, substance abuse may be preceded by reward seeking behaviors in childhood such that individuals who seek rewards, novelty, and display response navigational strategies at the expense spatial memory strategies during navigation, also have increased risks of neurological and psychiatric disorders associated with reduced gray matter in the hippocampus. Such individuals would benefit from a physical exercise program or stimulation of the frontal cortex, which have been shown to reduce drug seeking behavior. In addition, a cognitive training program designed to increase the use of hippocampal-based spatial strategies may benefit these individuals in combination with cognitive behavioural therapies aimed at reducing reward-seeking behaviors.

## References

[b1] Abdulla FA, Bradbury E, Calaminici MR, Lippiello PM, Wonnacott S, Gray JA, Sinden JD (1996). Relationship between up-regulation of nicotine binding sites in rat brain and delayed cognitive enhancement observed after chronic or acute nicotinic receptor stimulation. Psychopharmacology (Berl).

[b2] Abrous DN, Adriani W, Montaron MF, Aurousseau C, Rougon G, Moal Le M, Piazza PV (2002). Nicotine self-administration impairs hippocampal plasticity. J Neurosci.

[b3] Alvarez P, Zola-Morgan S, Squire LR (1995). Damage limited to the hippocampal region produces long-lasting memory impairment in monkeys. J Neurosci.

[b4] Amiaz R, Levy D, Vainiger D, Grunhaus L, Zangen A (2009). Repeated high-frequency transcranial magnetic stimulation over the dorsolateral prefrontal cortex reduces cigarette craving and consumption. Addiction.

[b5] Amico F, Meisenzahl E, Koutsouleris N, Reiser M, Moller HJ, Frodl T (2011). Structural MRI correlates for vulnerability and resilience to major depressive disorder. J Psychiatry Neurosci.

[b6] Apostolova LG, Dutton RA, Dinov ID, Hayashi KM, Toga AW, Cummings JL, Thompson PM (2006). Conversion of mild cognitive impairment to Alzheimer disease predicted by hippocampal atrophy maps. Arch Neurol.

[b7] Arias-Cavieres A, Rozas C, Reyes-Parada M, Barrera N, Pancetti F, Loyola S, Lorca RA, Zeise ML, Morales B (2010). MDMA (“ecstasy”) impairs learning in the Morris Water Maze and reduces hippocampal LTP in young rats. Neurosci Lett.

[b8] Banner H, Bhat V, Etchamendy N, Joober R, Bohbot VD (2011). The BDNF val66met polymorphism is associated with reduced fMRI activity in the hippocampus and increased use of caudate nucleus-dependent strategies in a human virtual navigation. Eur J Neurosci.

[b9] Barnes CA, Nadel L, Honig WK (1980). Spatial memory deficit in senescent rats. Can J Psychol.

[b10] Barrett SP, Pihl RO, Benkelfat C, Brunelle C, Young SN, Leyton M (2008). The role of dopamine in alcohol self-administration in humans: Individual differences. Eur Neuropsychopharmacol.

[b11] Baudonnat M, Guillou JL, Husson M, Vandesquille M, Corio M, Decorte L, Faugere A, Porte Y, Mons N, David V (2011). Disrupting effect of drug-induced reward on spatial but not cue-guided learning: Implication of the striatal protein kinase A/cAMP response element-binding protein pathway. J Neurosci.

[b12] Belin D, Deroche-Gamonet V (2012). Responses to novelty and vulnerability to cocaine addiction: Contribution of a multi-symptomatic animal model. Cold Spring Harb Perspect Med.

[b13] Bernal MC, Vicens P, Carrasco MC, Redolat R (1999). Effects of nicotine on spatial learning in C57BL mice. Behav Pharmacol.

[b14] Bohbot VD, Kalina M, Stepankova K, Spackova N, Petrides M, Nadel L (1998). Spatial memory deficits in patients with lesions to the right hippocampus and to the right parahippocampal cortex. Neuropsychologia.

[b15] Bohbot VD, Iaria G, Petrides M (2004). Hippocampal function and spatial memory: Evidence from functional neuroimaging in healthy participants and performance of patients with medial temporal lobe resections. Neuropsychology.

[b16] Bohbot VD, Lerch J, Thorndycraft B, Iaria G, Zijdenbos AP (2007). Gray matter differences correlate with spontaneous strategies in a human virtual navigation task. J Neurosci.

[b17] Bohbot VD, McKenzie S, Konishi K, Fouquet C, Kurdi V, Schachar R, Boivin M, Robaey P (2012). Virtual navigation strategies from childhood to senescence: Evidence for changes across the life span. Front Aging Neurosci.

[b18] Boileau I, Dagher A, Leyton M, Welfeld K, Booij L, Diksic M, Benkelfat C (2007). Conditioned dopamine release in humans: A positron emission tomography [11C]raclopride study with amphetamine. J Neurosci.

[b19] Brown TI, Ross RS, Keller JB, Hasselmo ME, Stern CE (2010). Which way was I going? Contextual retrieval supports the disambiguation of well learned overlapping navigational routes. J Neurosci.

[b20] Bueller JA, Aftab M, Sen S, Gomez-Hassan D, Burmeister M, Zubieta JK (2006). BDNF Val66Met allele is associated with reduced hippocampal volume in healthy subjects. Biol Psychiatry.

[b21] Castellanos-Ryan N, O'Leary-Barrett M, Sully L, Conrod P (2013). Sensitivity and specificity of a brief personality screening instrument in predicting future substance use, emotional, and behavioral problems: 18-month predictive validity of the Substance Use Risk Profile Scale. Alcohol Clin Exp Res.

[b22] Chang Q, Gold PE (2003). Switching memory systems during learning: Changes in patterns of brain acetylcholine release in the hippocampus and striatum in rats. J Neurosci.

[b23] Cheetham A, Allen NB, Whittle S, Simmons JG, Yucel M, Lubman DI (2012). Orbitofrontal volumes in early adolescence predict initiation of cannabis use: A 4-year longitudinal and prospective study. Biol Psychiatry.

[b24] Conrad CD, Galea LA, Kuroda Y, McEwen BS (1996). Chronic stress impairs rat spatial memory on the Y maze, and this effect is blocked by tianeptine pretreatment. Behav Neurosci.

[b25] Cosgrove KP, Hunter RG, Carroll ME (2002). Wheel-running attenuates intravenous cocaine self-administration in rats: Sex differences. Pharmacol Biochem Behav.

[b26] Cox SM, Benkelfat C, Dagher A, Delaney JS, Durand F, McKenzie SA, Kolivakis T, Casey KF, Leyton M (2009). Striatal dopamine responses to intranasal cocaine self-administration in humans. Biol Psychiatry.

[b27] Dahmani L, Bohbot VD (2013). Increased activity and grey matter in the orbitofrontal cortex associated with hippocampus-dependent spatial learning. Baycrest 23rd Annual Neuroscience Conference Abstracts.

[b28] Dickinson A, Wood N, Smith JW (2002). Alcohol seeking by rats: Action or habit?. Q J Exp Psychol B.

[b29] Driscoll I, Hamilton DA, Yeo RA, Brooks WM, Sutherland RJ (2005). Virtual navigation in humans: The impact of age, sex, and hormones on place learning. Horm Behav.

[b30] Eichenbaum H, Stewart C, Morris RG (1990). Hippocampal representation in place learning. J Neurosci.

[b31] Erickson KI, Boot WR, Basak C, Neider MB, Prakash RS, Voss MW, Graybiel AM, Simons DJ, Fabiani M, Gratton G, Kramer AF (2010). Striatal volume predicts level of video game skill acquisition. Cereb Cortex.

[b32] Erickson KI, Voss MW, Prakash RS, Basak C, Szabo A, Chaddock L, Kim JS, Heo S, Alves H, White SM (2011). Exercise training increases size of hippocampus and improves memory. Proc Natl Acad Sci USA.

[b33] Ersche KD, Barnes A, Jones PS, Morein-Zamir S, Robbins TW, Bullmore ET (2011). Abnormal structure of frontostriatal brain systems is associated with aspects of impulsivity and compulsivity in cocaine dependence. Brain.

[b34] Etchamendy N, Konishi K, Pike GB, Marighetto A, Bohbot VD (2012). Evidence for a virtual human analog of a rodent relational memory task: A study of aging and fMRI in young adults. Hippocampus.

[b35] Everitt BJ, Robbins TW (2005). Neural systems of reinforcement for drug addiction: From actions to habits to compulsion. Nat Neurosci.

[b36] Everitt BJ, Dickinson A, Robbins TW (2001). The neuropsychological basis of addictive behaviour. Brain Res Brain Res Rev.

[b137] Feil J, Zangen A (2010). Brain stimulation in the study and treatment of addiction. Neurosci Biobehav Rev.

[b37] Flagel SB, Robinson TE, Clark JJ, Clinton SM, Watson SJ, Seeman P, Phillips PE, Akil H (2010). An animal model of genetic vulnerability to behavioral disinhibition and responsiveness to reward-related cues: Implications for addiction. Neuropsychopharmacology.

[b38] Flagel SB, Clark JJ, Robinson TE, Mayo L, Czuj A, Willuhn I, Akers CA, Clinton SM, Phillips PE, Akil H (2011). A selective role for dopamine in stimulus-reward learning. Nature.

[b122] Foerde K, Shohamy D (2011). Feedback timing modulates brain systems for learning in humans. J Neurosci.

[b39] Gabriele A, Setlow B, Packard MG (2009). Cocaine self-administration alters the relative effectiveness of multiple memory systems during extinction. Learn Mem.

[b40] Gallinat J, Lang UE, Jacobsen LK, Bajbouj M, Kalus P, Haebler von D, Seifert F, Schubert F (2007). Abnormal hippocampal neurochemistry in smokers: Evidence from proton magnetic resonance spectroscopy at 3 T. J Clin Psychopharmacol.

[b41] Gilbertson MW, Shenton ME, Ciszewski A, Kasai K, Lasko NB, Orr SP, Pitman RK (2002). Smaller hippocampal volume predicts pathologic vulnerability to psychological trauma. Nat Neurosci.

[b42] Gold PE (2004). Coordination of multiple memory systems. Neurobiol Learn Mem.

[b43] Gray JA (1987). Perspectives on anxiety and impulsivity: A commentary. J Res Personality.

[b44] Groman SM, Morales AM, Lee B, London ED, Jentsch JD (2013). Methamphetamine-induced increases in putamen gray matter associate with inhibitory control. Psychopharmacology.

[b45] Hartley T, Maguire EA, Spiers HJ, Burgess N (2003). The well-worn route and the path less traveled: Distinct neural bases of route following and wayfinding in humans. Neuron.

[b46] Head D, Isom M (2010). Age effects on wayfinding and route learning skills. Behav Brain Res.

[b47] Ho YS, Yang X, Yeung SC, Chiu K, Lau CF, Tsang AW, Mak JC, Chang RC (2012). Cigarette smoking accelerated brain aging and induced pre-Alzheimer-like neuropathology in rats. PLoS One.

[b48] Iaria G, Petrides M, Dagher A, Pike B, Bohbot VD (2003). Cognitive strategies dependent on the hippocampus and caudate nucleus in human navigation: Variability and change with practice. J Neurosci.

[b49] Indlekofer F, Piechatzek M, Daamen M, Glasmacher C, Lieb R, Pfister H, Tucha O, Lange KW, Wittchen HU, Schutz CG (2009). Reduced memory and attention performance in a population-based sample of young adults with a moderate lifetime use of cannabis, ecstasy and alcohol. J Psychopharmacol.

[b50] Jager G, Hell van HH, Win de MM, Kahn RS, Brink van Den W, Ree van JM, Ramsey NF (2007). Effects of frequent cannabis use on hippocampal activity during an associative memory task. Eur Neuropsychopharmacol.

[b51] Kleen JK, Sitomer MT, Killeen PR, Conrad CD (2006). Chronic stress impairs spatial memory and motivation for reward without disrupting motor ability and motivation to explore. Behav Neurosci.

[b52] Koepp MJ, Gunn RN, Lawrence AD, Cunningham VJ, Dagher A, Jones T, Brooks DJ, Bench CJ, Grasby PM (1998). Evidence for striatal dopamine release during a video game. Nature.

[b143] Kühn S, Gallinat J (2013). Amount of lifetime video gaming is positively associated with entorhinal, hippocampal and occipital volume. Mol Psychiatry. 2013 Epub.

[b53] Kuhn S, Romanowski A, Schilling C, Lorenz R, Morsen C, Seiferth N, Banaschewski T, Barbot A, Barker GJ, Buchel C, Conrod PJ, Dalley JW, Flor H, Garavan H, Ittermann B, Mann K, Martinot JL, Paus T, Rietschel M, Smolka MN, Strohle A, Walaszek B, Schumann G, HeinzA GallinatJ (2011). The neural basis of video gaming. Transl Psychiatry.

[b54] Lansink CS, Goltstein PM, Lankelma JV, McNaughton BL, Pennartz CM (2009). Hippocampus leads ventral striatum in replay of place-reward information. PLoS Biol.

[b55] Lerch JP, Yiu AP, Martinez-Canabal A, Pekar T, Bohbot VD, Frankland PW, Henkelman RM, Josselyn SA, Sled JG (2011). Maze training in mice induces MRI-detectable brain shape changes specific to the type of learning. Neuroimage.

[b56] Lin SY, Calcott R, Germann J, Konishi K, Bohbot VD, Lerch JP (2012). Decreased use of hippocampus–dependent spatial strategy in favor of caudate nucleus-dependent response strategy from childhood to adolescence. Society for Neuroscience 2012 Abstracts.

[b57] London ED, Ernst M, Grant S, Bonson K, Weinstein A (2000). Orbitofrontal cortex and human drug abuse: Functional imaging. Cereb Cortex.

[b58] Lu G, Zhou QX, Kang S, Li QL, Zhao LC, Chen JD, Sun JF, Cao J, Wang YJ, Chen J, Chen XY, Zhong DF, Chi ZQ, Xu L, Liu JG (2010). Chronic morphine treatment impaired hippocampal long-term potentiation and spatial memory via accumulation of extracellular adenosine acting on adenosine A1 receptors. J Neurosci.

[b59] McDonald RJ, White NM (1993). A triple dissociation of memory systems: Hippocampus, amygdala, and dorsal striatum. Behav Neurosci.

[b60] McDonald RJ, White NM (1994). Parallel information processing in the water maze: Evidence for independent memory systems involving dorsal striatum and hippocampus. Behav Neural Biol.

[b61] McEwen BS, Sapolsky RM (1995). Stress and cognitive function. Curr Opin Neurobiol.

[b62] McKittrick CR, Magarinos AM, Blanchard DC, Blanchard RJ, McEwen BS, Sakai RR (2000). Chronic social stress reduces dendritic arbors in CA3 of hippocampus and decreases binding to serotonin transporter sites. Synapse.

[b63] McMillan DE, McClure GY, Hardwick WC (1995). Effects of access to a running wheel on food, water and ethanol intake in rats bred to accept ethanol. Drug Alcohol Depend.

[b64] Miles FJ, Everitt BJ, Dickinson A (2003). Oral cocaine seeking by rats: Action or habit?. Behav Neurosci.

[b65] Milivojevic D, Milovanovic SD, Jovanovic M, Svrakic DM, Svrakic NM, Svrakic SM, Cloninger CR (2012). Temperament and character modify risk of drug addiction and influence choice of drugs. Am J Addict.

[b66] Mizumori SJ, Yeshenko O, Gill KM, Davis DM (2004). Parallel processing across neural systems: Implications for a multiple memory system hypothesis. Neurobiol Learn Mem.

[b67] Noonan MA, Bulin SE, Fuller DC, Eisch AJ (2010). Reduction of adult hippocampal neurogenesis confers vulnerability in an animal model of cocaine addiction. J Neurosci.

[b68] O'Keefe J, Nadel L (1978). The hippocampus as a cognitive map.

[b69] Packard MG (1999). Glutamate infused posttraining into the hippocampus or caudate-putamen differentially strengthens place and response learning. Proc Natl Acad Sci USA.

[b70] Packard MG, McGaugh JL (1996). Inactivation of hippocampus or caudate nucleus with lidocaine differentially affects expression of place and response learning. Neurobiol Learn Mem.

[b71] Packard MG, Hirsh R, White NM (1989). Differential effects of fornix and caudate nucleus lesions on two radial maze tasks: Evidence for multiple memory systems. J Neurosci.

[b72] Pantelis C, Velakoulis D, McGorry PD, Wood SJ, Suckling J, Phillips LJ, Yung AR, Bullmore ET, Brewer W, Soulsby B, Desmond P, McGuire PK (2003). Neuroanatomical abnormalities before and after onset of psychosis: A cross-sectional and longitudinal MRI comparison. Lancet.

[b73] Pingault JB, Cote SM, Galera C, Genolini C, Falissard B, Vitaro F, Tremblay RE (2013). Childhood trajectories of inattention, hyperactivity and oppositional behaviors and prediction of substance abuse/dependence: A 15-year longitudinal population-based study. Mol Psychiatry.

[b74] Piri M, Nasehi M, Shahab Z, Zarrindast MR (2012). The effects of nicotine on nitric oxide induced anxiogenic-like behaviors in the dorsal hippocampus. Neurosci Lett.

[b75] Rapp PR, Kansky MT, Roberts JA (1997). Impaired spatial information processing in aged monkeys with preserved recognition memory. Neuroreport.

[b76] Rodgers MK, Sindone JA, Moffat SD (2012). Effects of age on navigation strategy. Neurobiol Aging.

[b77] Rubino T, Realini N, Braida D, Alberio T, Capurro V, Vigano D, Guidali C, Sala M, Fasano M, Parolaro D (2009). The depressive phenotype induced in adult female rats by adolescent exposure to THC is associated with cognitive impairment and altered neuroplasticity in the prefrontal cortex. Neurotox Res.

[b78] Sapolsky RM (1994). The physiological relevance of glucocorticoid endangerment of the hippocampus. Ann NY Acad Sci.

[b79] Sapolsky RM, Uno H, Rebert CS, Finch CE (1990). Hippocampal damage associated with prolonged glucocorticoid exposure in primates. J Neurosci.

[b80] Saunders BT, Robinson TE (2010). A cocaine cue acts as an incentive stimulus in some but not others: Implications for addiction. Biol Psychiatry.

[b81] Saunders BT, Robinson TE (2011). Individual variation in the motivational properties of cocaine. Neuropsychopharmacology.

[b82] Scerri C, Stewart CA, Breen KC, Balfour DJ (2006). The effects of chronic nicotine on spatial learning and bromodeoxyuridine incorporation into the dentate gyrus of the rat. Psychopharmacology (Berl).

[b83] Schwabe L, Oitzl MS, Philippsen C, Richter S, Bohringer A, Wippich W, Schachinger H (2007). Stress modulates the use of spatial versus stimulus-response learning strategies in humans. Learn Mem.

[b84] Schwabe L, Dalm S, Schachinger H, Oitzl MS (2008). Chronic stress modulates the use of spatial and stimulus-response learning strategies in mice and man. Neurobiology of Learning and Memory.

[b85] Schwabe L, Schachinger H, Kloet de ER, Oitzl MS (2010). Stress impairs spatial but not early stimulus-response learning. Behav Brain Res.

[b86] Schwabe L, Dickinson A, Wolf OT (2011). Stress, habits, and drug addiction: A psychoneuroendocrinological perspective. Exp Clin Psychopharmacol.

[b87] Schwabe L, Bohbot VD, Wolf OT (2012). Prenatal stress changes learning strategies in adulthood. Hippocampus.

[b89] Scoville WB, Milner B (1957). Loss of recent memory after bilateral hippocampal lesions. J Neurol Neurosurg Psychiatry.

[b189] Shohamy D, Adcock RA (2010). Dopamine and adaptive memory. Trends Cogn Sci.

[b90] Small DM, Zatorre RJ, Dagher A, Evans AC, Jones-Gotman M (2001). Changes in brain activity related to eating chocolate: From pleasure to aversion. Brain.

[b91] Smith MA, Pitts EG (2011). Access to a running wheel inhibits the acquisition of cocaine self-administration. Pharmacol Biochem Behav.

[b92] Smith MA, Schmidt KT, Iordanou JC, Mustroph ML (2008). Aerobic exercise decreases the positive-reinforcing effects of cocaine. Drug Alcohol Depend.

[b123] Smith-Dijak A, Randhawa J, Murty VP, Adcock RA, Bohbot VD (2013). Benefits of immediate financial rewards are selective to response learners but not spatial learners tested in a virtual navigation task.

[b93] Socci DJ, Sanberg PR, Arendash GW (1995). Nicotine enhances Morris water maze performance of young and aged rats. Neurobiol Aging.

[b94] Swan GE, Lessov-Schlaggar CN (2007). The effects of tobacco smoke and nicotine on cognition and the brain. Neuropsychol Rev.

[b95] Tanaka S, Ikeda H, Kasahara K, Kato R, Tsubomi H, Sugawara SK, Mori M, Hanakawa T, Sadato N, Honda M, Watanabe K (2013). Larger Right Posterior Parietal Volume in Action Video Game Experts: A Behavioral and Voxel-Based Morphometry (VBM) Study. PLoS One.

[b96] Tanapat P, Hastings NB, Rydel TA, Galea LA, Gould E (2001). Exposure to fox odor inhibits cell proliferation in the hippocampus of adult rats via an adrenal hormone-dependent mechanism. J Comp Neurol.

[b97] Tarter RE, Kirisci L, Mezzich A, Cornelius JR, Pajer K, Vanyukov M, Gardner W, Blackson T, Clark D (2003). Neurobehavioral disinhibition in childhood predicts early age at onset of substance use disorder. Am J Psychiatry.

[b98] Taylor AH, Ussher MH, Faulkner G (2007). The acute effects of exercise on cigarette cravings, withdrawal symptoms, affect and smoking behaviour: A systematic review. Addiction.

[b99] Vafaei AA, Rashidy-Pour A (2004). Reversible lesion of the rat's orbitofrontal cortex interferes with hippocampus-dependent spatial memory. Behav Brain Res.

[b100] Meer van der MA, Johnson A, Schmitzer-Torbert NC, Redish AD (2010). Triple dissociation of information processing in dorsal striatum, ventral striatum, and hippocampus on a learned spatial decision task. Neuron.

[b101] Praag van H (2008). Neurogenesis and exercise: Past and future directions. Neuromolecular Med.

[b102] Welch KA, Carson A, Lawrie SM (2013). Brain structure in adolescents and young adults with alcohol problems: Systematic review of imaging studies. Alcohol Alcohol.

[b103] White NM (1996). Addictive drugs as reinforcers: Multiple partial actions on memory systems. Addiction.

[b104] Wolbers T, Weiller C, Buchel C (2004). Neural foundations of emerging route knowledge in complex spatial environments. Brain Res Cogn Brain Res.

[b105] Yucel M, Solowij N, Respondek C, Whittle S, Fornito A, Pantelis C, Lubman DI (2008). Regional brain abnormalities associated with long-term heavy cannabis use. Arch Gen Psychiatry.

